# Supplementary material for: Development of a rapid and specific MALDI-TOF mass spectrometric assay for SARS-CoV-2 detection
Source: Clin Proteomics. 2023 Jul 1;20:26. doi: 10.1186/s12014-023-09415-y (PMC10314570; doi:10.1186/s12014-023-09415-y)
Supplement: Supplementary file 1 — Supplementary Material 1:Table S1. LC-MS/MS MRM (multiple reaction monitoring) method settings using the SARS-CoV-2 LC-MS Kit (RUO, Waters Corp). Figure S1. MALDI-TOF mass spectrum of tryptic peptides from SARS-CoV-nucleoprotein. Figure S2. MALDI-TOF mass spectrum of peptides C and D (antibody-enriched) from SARS-CoV-2 nucleoprotein. Figure S3. MALDI-TOF mass spectrum of peptides A and C (antibody-enriched) from SARS-CoV-2 nucleoprotein, spiked into Cobas PCR Medium. Figure S4. Comparison of SARS-CoV-2 quantification with peptides SIL-A and SIL-C. Figure S5. Quantification of SARS-CoV-2 nucleoprotein with peptide SIL-C. [file 12014_2023_9415_MOESM1_ESM.docx]

Supporting Information

Development of a Rapid and Specific MALDI-TOF Mass Spectrometric Assay for SARS-CoV-2 Detection

Lydia Kollhoff^1,2^, Marc Kipping^1,2^, Manfred Rauh^3^, Uta Ceglarek^4^, Günes Barka^5^, Frederik Barka^5^, Andrea Sinz^1,2*^

^1^Department of Pharmaceutical Chemistry and Bioanalytics, Martin Luther University Halle-Wittenberg, Halle (Saale), Germany; ^2^Center for Structural Mass Spectrometry, Martin Luther University Halle-Wittenberg, Halle (Saale), Germany; ^3^Department of Pediatrics and Adolescent Medicine, Friedrich Alexander University Erlangen-Nürnberg, Germany; ^4^Institute for Laboratory Medicine, Clinical Chemistry and Molecular Diagnostics, University of Leipzig, Leipzig, Germany; ^5^SunChrom Wissenschaftliche Geräte GmbH, 61381 Friedrichsdorf, Germany

*Address correspondence to: [andrea.sinz@pharmazie.uni-halle.de](mailto:andrea.sinz@pharmazie.uni-halle.de)

Department of Pharmaceutical Chemistry and Bioanalytics, Center for Structural Mass Spectrometry, Martin Luther University Halle-Wittenberg, Kurt-Mothes-Str. 3, D-06120 Halle (Saale), Germany

**Table of Contents**

**Table S1**. LC-MS/MS MRM (multiple reaction monitoring) method settings using the SARS-CoV-2 LC-MS Kit (RUO, Waters Corp).

**Figure S1.** MALDI-TOF mass spectrum of tryptic peptides from SARS-CoV-2 nucleoprotein.

**Figure S2.** MALDI-TOF mass spectrum of peptides C and D (antibody-enriched) from SARS-CoV-2 nucleoprotein.

**Figure S3.** MALDI-TOF mass spectrum of peptides A and C (antibody-enriched) from SARS-CoV-2 nucleoprotein, spiked into Cobas PCR Medium.

**Figure S4.** Comparison of SARS-CoV-2 quantification with peptides SIL-A and SIL-C.

**Table S1.** LC-MS/MS MRM (multiple reaction monitoring) method settings.

| **Peptide** | **MRM** |  | **Cone (V)** | **Collison (V)** | **Retention Time (min)** | **Scan Window (min)** |
| --- | --- | --- | --- | --- | --- | --- |
| D | 564.8>400.2 | Quantifier | 35 | 19 | 1.24 | 0-5.0 |
|  | 564.8>584.4 | Qualifier | 35 | 20 | 1.24 | 0-5.0 |
|  | 564.8>712.4 | Qualifier | 35 | 24 | 1.24 | 0-5.0 |
| C | 563.8>679.4 | Quantifier | 35 | 19 | 1.93 | 0-5.0 |
|  | 563.8>578.3 | Qualifier | 35 | 18 | 1.93 | 0-5.0 |
|  | 563.8>892.5 | Qualifier | 35 | 19 | 1.93 | 0-5.0 |
| B | 687.4>841.5 | Quantifier | 35 | 18 | 2.63 | 0-5.0 |
|  | 687.4>766.4 | Qualifier | 35 | 23 | 2.63 | 0-5.0 |
|  | 687.4>865.5 | Qualifier | 35 | 23 | 2.63 | 0-5.0 |


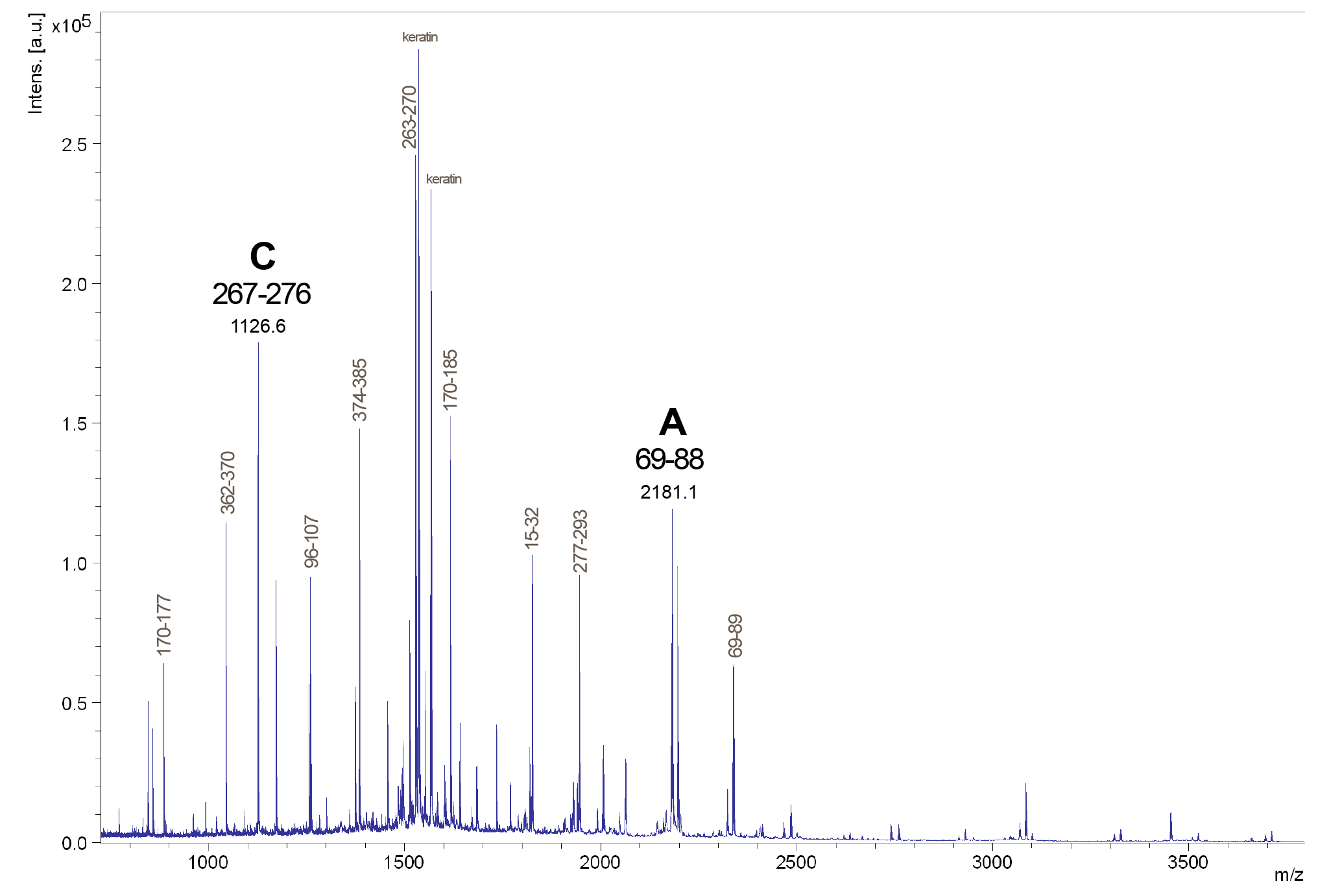


**Figure S1. MALDI-TOF mass spectrum of tryptic peptides from SARS-CoV-nucleoprotein**. Peptides from SARS-CoV-2 nucleoprotein are marked with their numbers in the amino acid sequence (see Figure 1). Peptides A and C are labeled.


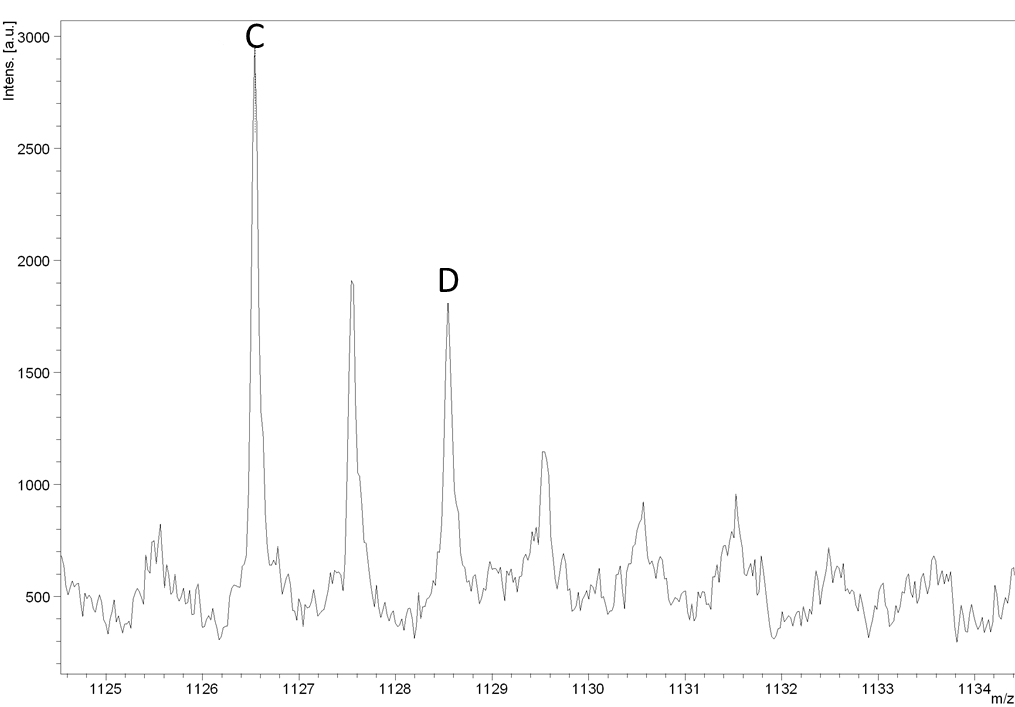


**Figure S2. MALDI-TOF mass spectrum of peptides C and D (antibody-enriched) from SARS-CoV‑2 nucleoprotein.** Signals of peptide D (ADETQALPQR), [M+H]^+^ at *m/z* 1128.6 and peptide C (AYNVTQAFGR), [M+H]^+^ at *m/z* 1126.6 are shown enlarged.

*
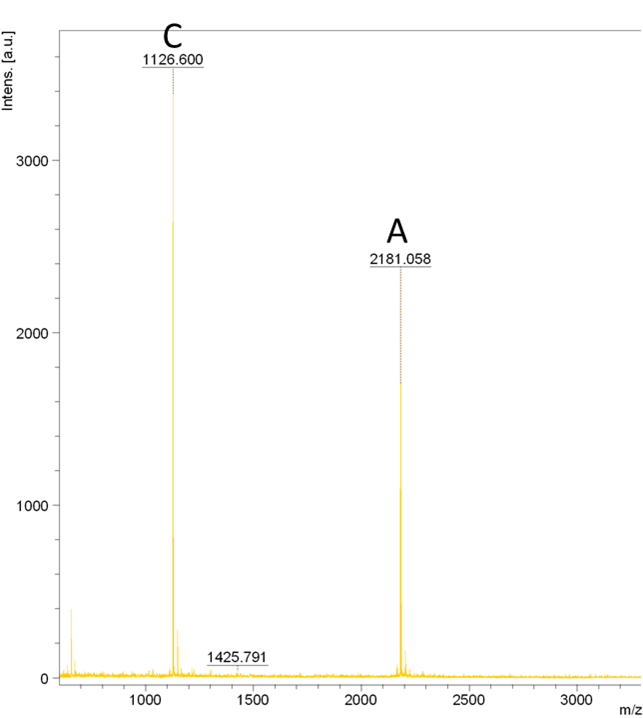
*

**Figure S3. MALDI-TOF mass spectrum of peptides A and C (antibody-enriched) from SARS‑CoV‑2 nucleoprotein, spiked into Cobas PCR Medium.** Peptides A and C are labeled.

*
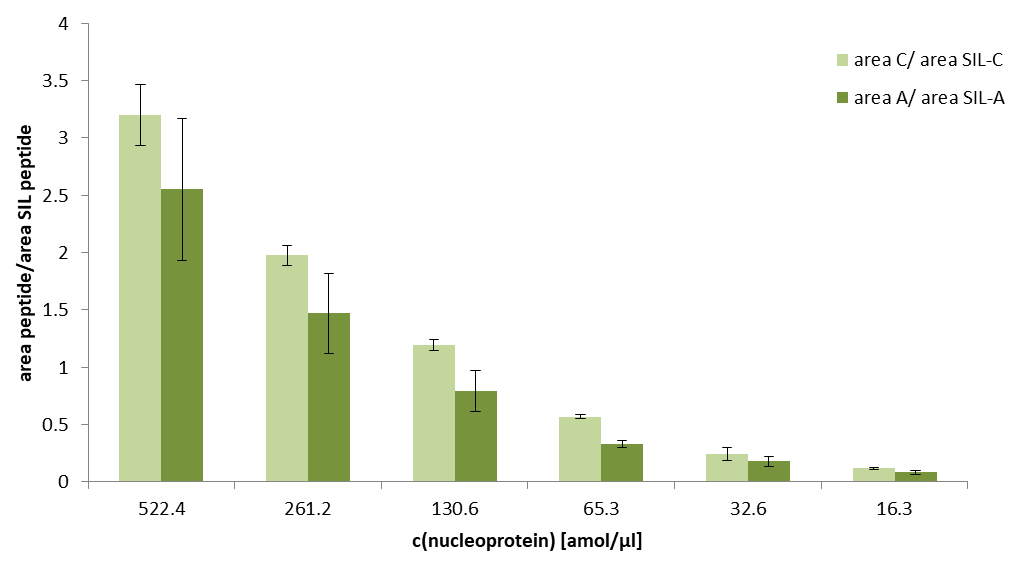
*

**Figure S4.** **Comparison of SARS-CoV-2 quantification with peptides SIL-A and SIL-C.** SARS‑CoV‑2 nucleoprotein was spiked at different concentrations (x-axis) into nasopharyngeal samples of healthy individuals (n=3). Samples were processed according to the workflow described in Figure 2 using MALDI-TOF-MS detection of peptides.
